# Chitosan-induced biotic stress tolerance and crosstalk with phytohormones, antioxidants, and other signalling molecules

**DOI:** 10.3389/fpls.2023.1217822

**Published:** 2023-07-19

**Authors:** Mohammad Mukarram, Jamin Ali, Hamed Dadkhah-Aghdash, Daniel Kurjak, František Kačík, Jaroslav Ďurkovič

**Affiliations:** ^1^ Department of Phytology, Faculty of Forestry, Technical University in Zvolen, Zvolen, Slovakia; ^2^ Centre for Applied Entomology and Parasitology, School of Life Sciences, Keele University, Newcastle-under-Lyme, Staffordshire, United Kingdom; ^3^ Department of Plant Biology, Faculty of Biological Sciences, Tarbiat Modares University, Tehran, Iran; ^4^ Department of Integrated Forest and Landscape Protection, Faculty of Forestry, Technical University in Zvolen, Zvolen, Slovakia; ^5^ Department of Chemistry and Chemical Technologies, Faculty of Wood Sciences and Technology, Technical University in Zvolen, Zvolen, Slovakia

**Keywords:** chitosan, biopolymer, antimicrobial, insecticidal, oxidative stress, phytohormones, antioxidants, chitooligosaccharides

## Abstract

Several polysaccharides augment plant growth and productivity and galvanise defence against pathogens. Such elicitors have ecological superiority over traditional growth regulators, considering their amplified biocompatibility, biodegradability, bioactivity, non-toxicity, ubiquity, and inexpensiveness. Chitosan is a chitin-derived polysaccharide that has recently been spotlighted among plant scientists. Chitosan supports plant growth and development and protects against microbial entities such as fungi, bacteria, viruses, nematodes, and insects. In this review, we discuss the current knowledge of chitosan’s antimicrobial and insecticidal potential with recent updates. These effects are further explored with the possibilities of chitosan’s active correspondence with phytohormones such as jasmonic acid (JA), salicylic acid (SA), indole acetic acid (IAA), abscisic acid (ABA), and gibberellic acid (GA). The stress-induced redox shift in cellular organelles could be substantiated by the intricate participation of chitosan with reactive oxygen species (ROS) and antioxidant metabolism, including hydrogen peroxide (H_2_O_2_), superoxide dismutase (SOD), catalase (CAT), and peroxidase (POD). Furthermore, we propose how chitosan could be intertwined with cellular signalling through Ca^2+^, ROS, nitric oxide (NO), transcription factors (TFs), and defensive gene activation.

## 1 Introduction

**Figure 1:**
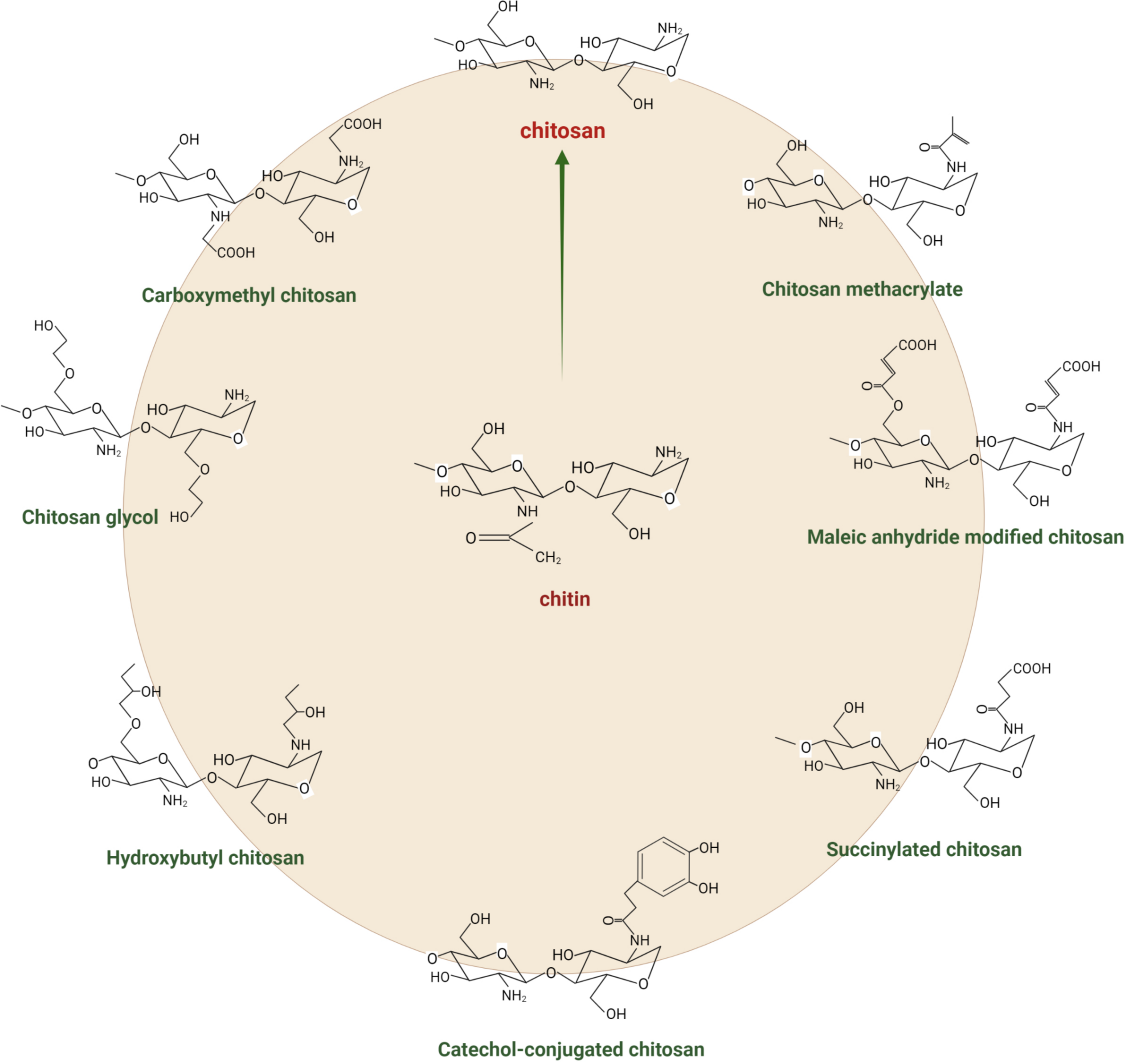
Chemical structure of chitin, chitosan, and its derivatives.

Chitosan is a polysaccharide derived from the second most abundant natural biopolymer on Earth, i.e., chitin. It is a linear cationic polysaccharide that is made up of β-(1,4)-joined *N*-acetyl-d-glucosamine (GlcNAc) and d-glucosamine (GLcN) units. This biopolymer is obtained commercially by the *N*-deacetylation of chitin from the crustacean exoskeleton. Some mushrooms, green algae, and yeasts can also biosynthesise it. The chemical structure of chitosan can be determined through three characteristics: the degree of polymerisation (the length of the polymer), the degree of acetylation (the percentage of acetylated units), and the pattern of acetylation (the sequence of GLcN and GlcNAc units) (Shokri et al., 2021). The chemical properties of chitosan depend on the degree of deacetylation (DDA), i.e., the extent to which amine groups have substituted *N*-acetyl groups in chitin (Mukarram et al., 2022). The more deacetylated the product, the more positive the charges and the higher the solubility under acidic conditions. The less deacetylated the chitosan, the higher the solubility under neutral and alkaline conditions (Aranaz et al., 2021; Linhorst et al., 2021). **Figure 1** depicts the chemical structure of chitosan and its common derivatives. Chitosan has several excellent chemical and physical properties that make it useful for many applications, e.g., biocompatibility, biodegradability, and antibacterial activity. These traits have led to using chitosan in various areas, including agriculture, food science, medicine, paper science and technologies, and environmental sciences (Morin-Crini et al., 2019). It also is used as a chelating agent because of its ability to bind with cholesterol, fats, proteins, and metal ions. In agriculture, chitosan has been used as a natural pesticide and plant growth agent due to its ability to improve plant growth and tolerance to environmental stresses. It effectively increases the yield and quality of crops such as vegetables, fruit, and ornamental plants. Chitosan has been used as a wound-healing promoter in medicine due to its ability to stimulate tissue regeneration and antibacterial properties (Croisier and Jérôme, 2013; Wang et al., 2020). In the food industry, chitosan has been used as a food stabiliser due to its ability to prevent food spoilage and improve the texture and stability of food products. In general, chitosan’s unique chemical structure and properties make it a versatile and valuable material with many applications (Ibrahim and El-Zairy, 2015; Rahman and Goswami, 2021; Shahrajabian et al., 2021; Gal et al., 2023).

Chitosan has been shown to act as a growth elicitor in plants, meaning that it can stimulate plant growth and improve crop yields. It could be due to the ability of chitosan to mimic the effects of plant growth regulators such as auxins and stimulate biosynthesis of other regulators such as ethylene (Pichyangkura and Chadchawan, 2015). In addition, chitosan has been shown to improve plant tolerance to environmental stresses such as drought, extreme temperatures, salinity, and heavy metal toxicity by enhancing the plant’s antioxidant defence system (Hidangmayum et al., 2019). The effectiveness of chitosan as a plant growth elicitor is significantly correlated with its chemical structure. The degree of polymerisation, degree of acetylation, the pattern of acetylation, and monomer unit sequences are all essential structural factors (Hosseinnejad and Jafari, 2016; Fan et al., 2023). Applying chitosan to plant roots, leaves, or seeds can increase plant height, root length, biomass production, and the improvement in crop yields. Chitosan has been shown to improve crop quality, including increased fruit size, vitamin and mineral content, and shelf life (Chamnanmanoontham et al., 2015; Faqir et al., 2021). In addition, it has also been used in the bioremediation of contaminated soils, as it can help to decrease the levels of heavy metals and other toxic ingredients in the soil. In general, the ability of chitosan to act as a growth elicitor in plants makes it a valuable compound for improving crop yields and quality and promoting sustainable agricultural methods (Faqir et al., 2021; Ingle et al., 2022; Kugarajah et al., 2023).

## 2 Chitosan against plant pathogens and pests

Chitosan has been used as an artificial plant defence elicitor for over two decades. It has been tested in various economically essential crops against various plant pathogens (Rabea et al., 2003). Many laboratory studies have investigated the correspondence of chitosan with pathogens and showed that it has a critical role in the triggering plant defence against microbes (fungi, oomycetes, bacteria, and viruses), nematodes, and insect herbivores (El Hadrami et al., 2010; Badawy and Rabea, 2011; Hadwiger, 2013). Chitosan treatment induces several protective reactions inhibiting pathogens spread and providing systemic acquired resistance to plants (Rabea et al., 2003; Chakraborty et al., 2020), including biosynthesis of phytoalexins (soybean) and lignification (antibiotic and antifeeding compounds in plants) (Horbowicz et al., 2009). As discussed, the antimicrobial activity of chitosan depends upon several factors such as degree of polymerisation, type of chitosan (native or modified), the host, pH, molecular weight (MW), degree of acetylation, and climatic conditions (Tsai and Su, 1999). Several studies suggest that chitosan’s pentamers and heptamers show higher antifungal activity than larger ones (Rabea et al., 2003). In other forms, antipathogenic activity increases with MW (Kulikov et al., 2006).

Chitosan-induced plant protection can be broadly categorised into direct and indirect defence. Direct defence affects the performance of attacking pathogens, including reduced growth rate (El Hadrami et al., 2010), lower fecundity (larviposition) (Haas et al., 2018), and higher mortality of pests in plants treated with chitosan and its derivatives (Ibrahim et al., 2022), while indirect defence shows enhanced recruitment of natural enemies (Naree et al., 2021; El-Seedi et al., 2022). The chitosan-induced direct defence could be due to the production of antibiotic and defensive compounds, such as reactive oxygen species (ROS), phytoalexins, and phenolic acid (Yin et al., 2012; Waewthongrak et al., 2015; Singh et al., 2017), which affect the physiological status of plant pathogens and emit plant volatiles to repel herbivores (Walling, 2000; Zhang and Chen, 2009).

Moreover, chitosan-treated plants have been found to release a qualitatively and quantitatively different blend of volatile organic compounds (VOCs) compared to untreated plants (Zhang and Chen, 2009; Badiali et al., 2018). The change in quality and quantity of VOCs depends on the concentration and duration of the induced phase of chitosan application (Yin et al., 2012). For instance, chitosan treatment of crop plants altered VOC emission, enhancing indirect plant defence by repelling and/or recruiting the natural enemies of insect herbivores (Obara et al., 2002). In particular, rice leaves treated with chitosan release a high amount of linalool, methyl salicylate (MeSA), (*Z*)-3-hexen-1-ol, and β-caryophyllene (Cartwright et al., 1977; Bailey, 1982; Kodama et al., 1992; Obara et al., 2002). Exogenous application of chitosan elicits expression of defence-related genes (1-deoxy-d-xylulose 5-phosphate synthase (DXS), PaDXS2B, and PaDXS2B) in seedlings of *Picea glauca* (Lapointe et al., 2001). Similarly, tomato plants treated with chitosan show higher levels of jasmonic acid (JA) than untreated plants (Doares et al., 1995). Encapsulation of geraniol in chitosan controls whitefly (De Oliveira et al., 2018). Chitosan induces resistance in *Solanum tuberosum* against late blight by accumulating salicylic acid (SA) caused by activation of benzoate-2-hydroxylase and hydrolysis of SA conjugates in *S. tuberosum* (Ozeretskovskaya et al., 2006) (**Table 1**).

**Table 1:** Chitosan-induced tolerance against pathogens and pests in higher plants.

Targeted pathogens	Observed effects	Species	Chitosan			References
			Type	Conc.	Method	
Fungi						
*Alternaria alternata*	Inhibition of mycelial growth and spore germination. Lesion formation was <50% in chitosan-treated fruit compared to control fruit. Chitosan treatment also inhibits the production of oxalic and fumaric acid in the host plant.	Mango (*Mangifera indica*) Potato (*Solanum tuberosum*)	Chitosan (1.74 × 10^4^ Da) 70%–75% degree of deacetylation Chitosan (shrimp shell)	1% conc. 10 g/L	Exogenous application Stem scar treatment	(López-Mora et al., 2013) (Reddy et al., 2000)
*Macrophomina phaseolina*	Inhibition of mycelial growth and spore germination.	Jute (*Corchorus olitorius*)	Water-soluble (s-chitosan, crab shell) (>85% deacetylation)	12.5 g/L	Hand spray	(Chatterjee et al., 2014)
*Rhizoctonia solani*	Inhibition of mycelial growth and spore germination.	Rice (*Oryza sativa*)	Chitosan (acid soluble) (degree of >85% deacetylation)	10 mg/mL	Added in medium (potato dextrose agar (PDA))	(Liu et al., 2012)
*Alternaria kikuchiana* Tanaka and *Physalospora piricola* Nose	Inhibitory effect on mycelial growth and spore germination. In the host plant, chitosan increases chitinase, β-1,3-glucanase and peroxidase activities.	Pear (*Pyrus communis*)	Chitosan (350 kDa) (90% deacetylation)	5 g/L	Added in medium (potato dextrose agar (PDA))	(Meng et al., 2010)
*Cylindrocladium floridanum*, *Cylindrocladium destructans*, *Fusarium acuminatum*, and *Fusarium oxysporum*	Chitosan reduces the radial growth of all these fungi and causes alterations such as increased vacuolation, cell wall thickening, hyphal distortion, retraction, and alteration of the plasma membrane.	Forest nurseries	Chitosan	–	Added in media PDA	(Laflamme et al., 2000)
*Fusarium solani*	Induces disease resistance protein in host plants and suppresses the germination and growth of fungal pathogens.	Pea (*Pisum sativa*)	Chitosan (snow crab shell) 80% deacetylation	1% w/v	Exogenous application on immature pea pods	(Hadwiger and Beckman, 1980)
*Botrytis cinerea*, *Rhizopus stolonifer*	Chitosan inhibits radial growth, spore germination, and germ tube elongation. These changes lead to the reduction of fruit decay caused by fungal pathogens.	Strawberry fruit (*Fragaria* × *ananassa*)	Chitosan (crab shell)	15 mg/L	Applied as fruit coatings	(El Ghaouth et al., 1992)
Virus						
Potato virus X, tobacco mosaic and necrosis viruses, alfalfa mosaic virus, peanut stunt virus, cucumber mosaic virus Potato virus X (PVX)	Inhibit the systemic propagation of viral/viroid infection. Accumulation of the virus was less on treated leaves. The resistance may be due to callose content and ribonuclease induction.	Bean (*Glycine max*), pea (*P. sativa*), tobacco (*Nicotiana tabacum*), tomato (*Lycopersicum esculentum*) Potato (*S. tuberosum*)	Chitosan (3, 36, 120 kDa) 85% deacetylation	1 mg/mL	Exogenous application	(Pospieszny et al., 1991; Pospieszny, 1997; Chirkov et al., 2001; Faoro et al., 2001; Struszczyk, 2002)
Bean mild mosaic virus (*Phaseolus vulgaris*)	Low-molecular-weight chitosan inhibits virus accumulation and systemic propagation.	Bean (*P. vulgaris*)	Chitosan (70 kDa) 85% deacetylation	100 µg/mL	Spray	(Kulikov et al., 2006)
Bacteria						
*Escherichia coli*, *Staphylococcus aureus*, and *Bacillus* species *Pseudomonas aeruginosa*, *Salmonella typhimurium* *Staphylococcus simulans*	Inhibit the growth, especially in acidic media, and rupture the cell membrane. Weaken barrier properties of the outer membrane of these bacteria. Leakage of UV-absorbing substances (likely nucleotide and coenzyme pools) and ultrastructural changes in the cell (cell wall giving rise to ‘vacuole-like’) structure.	Culture Luria-Bertani broth Standard I nutrient broth	Chitosan hydroglutamate Chitosan (250 ppm; crab shell) 85% deacetylation Chitosan (50–190 kDa) 70%–85% deacetylation	0.1 mg/mL 1 mg/mL 1% w/v	Added to the cell suspension Added to the cell suspension Added to the cell suspension	(Sudarshan et al., 1992; Kim et al., 1997; Helander et al., 2001; Jia and Xu, 2001; Liu et al., 2004) (Raafat et al., 2008)
Nematodes						
*Bursaphelenchus xylophilus*	Reduced the number of nematodes up to sevenfold on the treated plant.	Pine (*Pinus pinaster*)	Chitosan (acid-soluble) (310–375 kDa); 75% deacetylation	2% w/w	Added in substrate	(Nunes da Silva et al., 2014)
*Meloidogyne incognita*	Induced local and systemic resistance and accumulates phytoalexins in the tissue of host plants.	Potato (*S. tuberosum*) Tomato (*L. esculentum*)	Chitosan (water-soluble crab chitosan, 5 kDa)	0.01–3000 µg/mL	Potato tubers and tomato seeds treatment	(Vasyukova et al., 2001)
Soybean cyst nematodes (SCNs); *Heterodera glycines*	Combining nematophagous fungi and chitosan treatment suppresses the egg density of SCNs and enhances the efficacy of nematophagous fungi, *Hirsutella minnesotensis*.	Soybean (*G. max*)	Chitosan	1%	Mixed with soil	(Mwaheb et al., 2017)
*M. incognita*	Low-molecular-weight chitosan (2.27 × 10^5^ g/mol) with soil significantly reduced the population, egg mass, and root galling of root-knot nematode.	Tomato (*L. esculentum*)	Chitosan (acid-soluble, 2.27 × 10^5^ g/mol), 89% deacetylation	500 mg/mL	Mixed with soil/larvae treatment	(Khalil and Badawy, 2012)
*Meloidogyne javanica*	Increased appressorium differentiation in *Pochonia chlamydosporia* also enhances *P. chlamydosporia* parasitism of root-knot nematode’s egg.	Tomato (*Solanum lycopersicum*)	Chitosan (70 kDa), 85% deacetylation	2 mg/mL	Irrigation	(Escudero et al., 2017)
*B. xylophilus*	Chitosan application increases plant tolerance to pinewood nematode by promoting antioxidative metabolism in the host plant.	Pine (*P. pinaster*)	Chitosan (327 kDa) deacetylation degree ≥75%	4.4%	Exogenous application	(Nunes da Silva et al., 2021)
*Aphelenchoides besseyi*	Chitosan (concentration; 0.25%) treatment of seed was an effective control method to control white tip nematode.	Rice (*O. sativa*)	Chitosan	0.25%	Seed treatment	(Ibrahim and Kurniawati, 2020)
Insects						
Lepidoterans; *Helicoverpa armigera*, *Plutella xylostella* *Hemipterans* (aphids); *Rhopalosiphum padi*, *Sitobian aveane*, *Metopophium dirhodum*, *Myzus persicae*, *Hyalopterus prun*, *Aphis gossypii*	Chitosan was significantly effective against lepidopterans and hemipterans insect pests, with 80% mortality.	Larvae were placed on cole leaf	Chitosan (300 kDa) (80%) deacetylation	3 g/L	Hand sprayer	(Zhang et al., 2003)
*Tuta absoluta*	Chitosan nano formulation reduces the infestation of tomato pinworms.	Tomato (*Lycopersicon esculentum*)	Chitosan	10, 25, and 50 ppm	Exogenous application	(Sabbour and Solieman, 2016)
*Spodoptera littoralis*	Chitosan treatment ceased the feeding behaviour of larvae after 2–3 days, leading to 100% mortality.	Cauliflower (*Brassica oleracea* L.)	Chitosan (crab shell, 85% degree of deacetylation)	5 g/kg	Added in artificial diet	(Rabea et al., 2005)
*A. gossypii* *Callosobruchus maculatus*	The number of egg deposition/females on treated plants significantly decreased compared to the control.	Soybean (*G. max*)	Nano chitosan (1,000 ppm)	25%	Added in media PDA	(Sahab et al., 2015)
*Solenopsis invicta*	It causes morphological changes and decreases digestive enzyme activity in red ants’ midgut, enhancing the mortality rate.	A plastic container was used to perform the experiment	Chitosan (degree of 90% deacetylation)	0.3%	Added into suspension	(Zheng et al., 2021a)

### 2.1 Against fungi and oomycetes

In an increasing population, the control of fungal plant disease has become vital to meet food supply needs. Phytopathogenic fungi are the most common plant pathogens and cause many severe diseases. Over time, the widespread use of synthetic fungicides to combat fungal crop diseases has increased resistance to fungal pathogens (Bagheri et al., 2019). Most vegetable diseases are due to fungal pathogens (Koike et al., 2007). The role of chitosan as a biocontrol agent against fungal pathogens has been studied extensively (Abbey et al., 2019; dos Santos Gomes et al., 2021; Riseh et al., 2023). Chitosan inhibits mycelial growth, sporulation, spore viability, and germination of the fungal pathogens, probably due to chitosan’s ability to bind DNA to inhibit RNA synthesis in the target organism (Roller and Covill, 1999). Chitosan imposes fatalities against other fungi and oomycetes, such as *Phytophthora cinnamomi*, *Phytophthora palmivora*, *Gremmeniella abietina*, *Cryphonectria parasitica*, and *Heterobasidion annosum* (Kuyyogsuy et al., 2018; Silva-Castro et al., 2018; Matei et al., 2020). Chitosan protected the *S. tuberosum* plant against late blight disease by eliciting the induced systemic resistance, pattern recognition receptors, and several other defence-related genes, hormones, and enzymes (Zheng et al., 2021b). Furthermore, it can restrict mycelial growth and spore germination by regulating several genes of metabolism, cell membrane structure and function, and ribosome biogenesis (Huang et al., 2021). Chitosan and its derivatives attained a growth inhibition of up to 100% against *Phytophthora cambivora* and significantly reduced the mycelial growth in *Phytophthora plurivora* and *Phytophthora* × *alni* (Silva-Castro et al., 2018). It could result from enhanced activities of catalase (CAT), peroxidase (POD), polyphenol oxidase, and phenylalanine ammonia lyase (PAL) and the expression of *HbPR1*, *HbGLU*, *HbASI*, and *HbCAT* genes with chitosan application (Kuyyogsuy et al., 2018). The same study found a positive correlation between chitosan and callose and lignin depositions in *Hevea brasiliensis* leaves, which could provide additional fungal resistance. Furthermore, Guo et al. (2013) reported that 0.5 mg/mL of chitosan reduced growth in *Fusarium oxysporum* (15%) and *Alternaria solani* (57%). Similar effects were observed by Younes et al. (2014), where chitosan effectively suppressed the growth of *Aspergillus niger*, *F. oxysporum*, and *A. solani*.

### 2.2 Against bacteria

The antibacterial activity of chitosan encompasses both discouraging bacterial growth (bacteriostatic) and destroying them (bactericidal). Although the mode of chitosan activity against bacteria is still under discussion, the most accepted model is the electrostatic interaction model governing chitosan binding to the bacterial membrane. The functional NH_2_ groups of GlcNAc subunits give chitosan a polycationic nature. The positive moiety binds electrostatically with the negatively charged components of bacterial cell membrane including extracellular polymeric substances and proteins (Khan et al., 2020). A few studies noted that chitosan can also bind to DNA and cause extensive nucleic acid degradation (Dananjaya et al., 2016; Wang et al., 2022). Chitosan binding induces permeabilisation of the bacterial cell surface and facilitates intracellular leakage. It degrades bacterial biofilm and causes cell death. However, it all depends on how well chitosan can perforate the plasma membrane, which directly depends on its MW and the bacterial type (Verlee et al., 2017). Several other factors can regulate chitosan’s antibacterial potential, such as the ratio of its monomeric units (GlcNAc and GLcN), the DDA, the acetylation pattern, solubility, and environmental effects (Li and Zhuang, 2020). Nonetheless, no substantial relation was found between the source of chitosan to its antibacterial activity. It is observed in several gram (+) and gram (−) bacteria that the minimum inhibitory concentration (MIC) of chitosan (similar MW) decreases with increasing DDA (Verlee et al., 2017). Thus, a higher DDA would mean more effective chitosan against bacteria. Antibacterial activity of chitosan has been noted against several important gram (+) bacteria (such as *Staphylococcus aureus*, *Bacillus cereus*, *Enterococcus faecalis*, *Micrococcus luteus*, and *Bacillus subtilis*) and gram (−) bacteria (including *Escherichia coli*, *Vibrio cholera*, *Shigella dysenteriae*, *Bacteroides fragilis*, and *Pseudomonas aeruginosa*) (Benhabiles et al., 2012; Younes et al., 2014; Goy et al., 2016; Yadav et al., 2020).

### 2.3 Against viruses

Plant viruses negatively impact plants and cause a wide range of symptoms, including discolouration, distortion of plant parts, and loss of vigour, affecting yield. Chitosan application has been found significantly effective against several viruses, including potato virus X, tobacco mosaic and necrosis viruses, alfalfa mosaic virus, peanut stunt virus, cucumber mosaic virus, potato virus X (PVX), and beans mild mosaic virus (*Phaseolus vulgaris*) (Pospieszny et al., 1991; Pospieszny, 1997; Chirkov et al., 2001; Faoro et al., 2001). Since chitosan’s antiviral activity depends on its MW, low MW chitosan shows higher antiviral activities than high MW. Chitosan application inhibits systemic propagation of viroid (Pospieszny et al., 1991) and induces plant resistance by callose formation and enhanced ribonuclease content. Treated leaves show less accumulation of viruses than the control (Chirkov et al., 2001).

### 2.4 Against nematodes

The underground plant parasitic nematodes attack various crops’ root systems, causing root galls, stunted growth, and increased susceptibility to pathogen attack and abiotic stress (Yan and Xie, 2015). Accumulating body of evidence has shown that exogenous application of chitosan triggers plant defence-related pathways in various crops, such as potato, tomato (Vasyukova et al., 2001; Fan et al., 2020; Boamah et al., 2023), barley, banana (Maciá-Vicente et al., 2009; Suarez-Fernandez et al., 2021), and soybean (Mwaheb et al., 2017). Consistently, chitosan also promotes host resistance to *Meloidogyne* spp. root-knot nematodes (RKNs). Similarly, water-soluble chitosan induces resistance against root-knot nematodes (Escudero et al., 2017; Chakraborty et al., 2020).

### 2.5 Against insects

Insect herbivores are among the most notorious crop pests that cause significant direct and indirect crop loss. The exogenous application of chitosan has been found effective against multiple pests, including chewing and sucking insects. The insecticidal effect of chitosan has been tested against various insect herbivores: lepidopterans (*Helicoverpa armigera*, *Plutella xylostella*, *Spodoptera littoralis*, and *Tuta absoluta*), hemipterans (*Aphis gossypii*, *Rhopalosiphum padi*, *Sitobian aveane*, *Metapophium dirhodum*, *Myzus persicae*, and *Hyalopetrus prun*), coleopterans (*Callosobruchus maculatus*), and hymenopterans (*Solenopsis invicta*) (Rabea et al., 2005; Sahab et al., 2015; Sabbour and Solieman, 2016; Zhang et al., 2003; Zhu et al., 2021). Chitosan-mediated changes have been shown to reduce egg deposition (Sahab et al., 2015), insect performance (Zhang et al., 2003), population densities (Sabbour and Solieman, 2016), and feeding behaviour. It also causes morphological changes in the midgut of insect herbivores and reduces digestive enzymes’ activity, increasing pest mortality (Rabea et al., 2005).

## 3 How does chitosan work?

**Figure 2:**
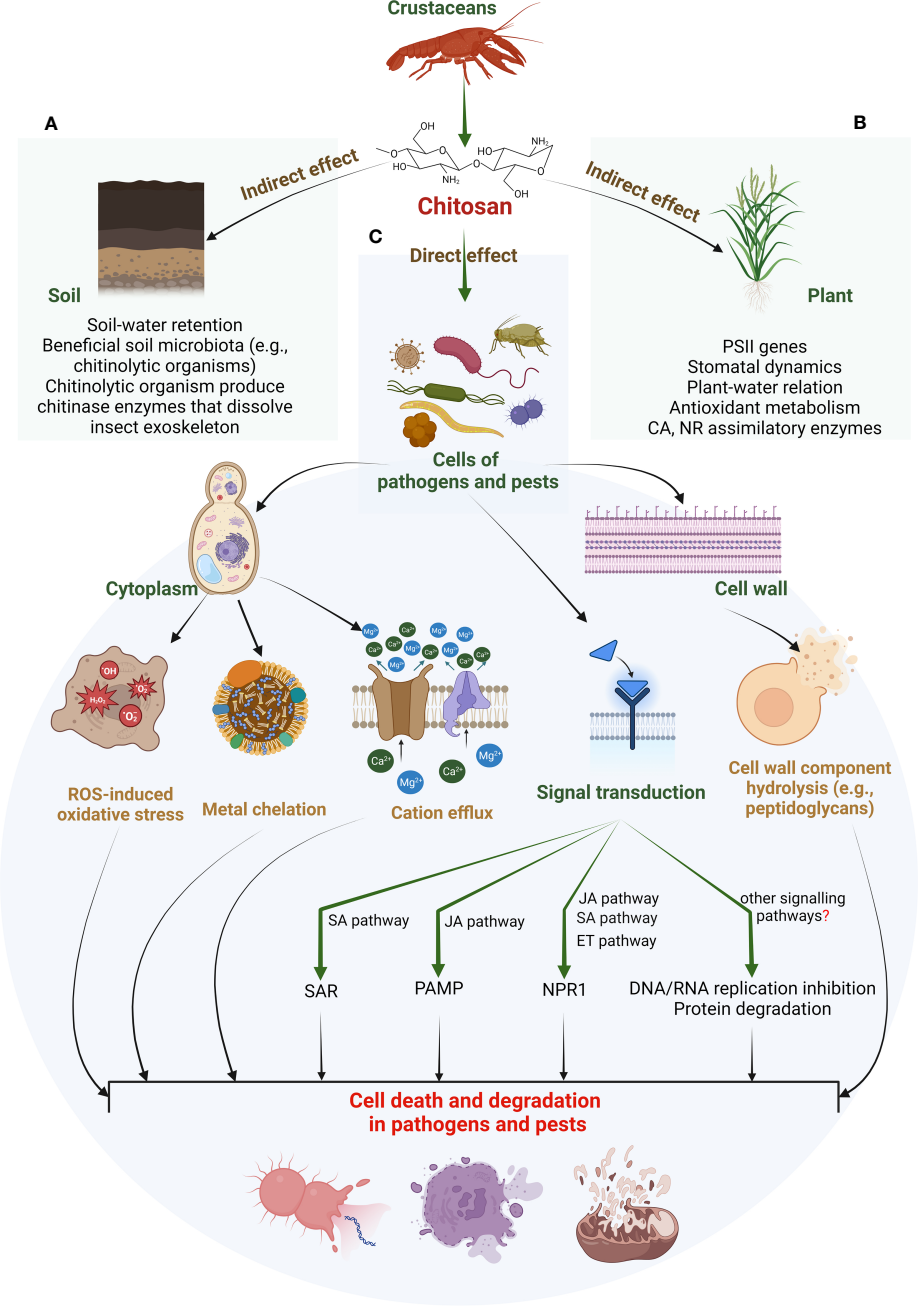
Proposed *modus operandi* for chitosan-induced biotic stress tolerance in higher plants. These effects can be identified as indirect **(A, B)** and direct **(C)**. Indirect effects include chitosan action on soil properties that improve water retention. Improved soil–water content promotes soil microbiota. Such microbiota include several chitinolytic organisms that can dissolve insect exoskeletons by chitinase enzymes (see panel **A**). Other beneficial soil organisms can promote source-sink potential. A higher nutrient status combined with increased soil–water retention promotes plant innate defence, e.g., antioxidant capacity (see panel **B**). In contrast, the direct effects of chitosan include cytotoxic action on pathogen/pest cells. Chitosan can cause hydrolysis of the cell wall components. This, along with metal chelation and cation efflux from the cytoplasm, disrupts ROS-antioxidant metabolism in pathogen/pest cells. This could result in cell degradation or death of plant invaders. It is suggested that these direct actions can rely on chitosan’s intricate crosstalk with certain phytohormones such as JA, SA, ET, ABA, and GA. A few studies suggested a genomic approach for such adjustments such as activation of defensive genes and TFs (see panel **C**). Apart from this, other signalling pathways could be facilitating chitosan-induced biotic tolerance in plants. Nevertheless, their understanding is still in preliminary phase and demands more investigation. PSII, photosystem II; CA, carbonic anhydrase; NR, nitrate reductase; ROS, reactive oxygen species; SAR, systemic induced resistance; PAMP, pathogen-associated molecular pattern; NPR1, natriuretic peptide receptor A/guanylate cyclase A; SA, salicylic acid; JA, jasmonic acid; ET, ethylene; GA, gibberellic acid; ABA, abscisic acid; TFs, transcription factors.

There are several theories regarding the antimicrobial mechanism of chitosan. Goy et al. (2009) proposed three faces of the antibacterial mechanism of chitosan: degradation of the cell wall by ionic surface interaction, inhibition of protein and mRNA synthesis through permeation of chitosan into nuclei of microorganisms, and limitation of nutrient availability for microorganism by the formation of external covering over the plant surface. Another researcher stated that the mechanism of chitosan action is based on the destruction of the cell membrane due to a burst of extracellular components, which has been observed in disrupting the growth of fungi (Xing et al., 2015). Recent studies suggested that chitosan is responsible for the hydrolysis of cell wall components (peptidoglycans), disrupting electrolytic balance and increasing pathogen mortality. The exogenous spray of chitosan induces resistance against insect herbivores. Chitosan has been extensively exploited to improve inducible plant defences against insect herbivores (Gatehouse, 2002). Chitosan treatment enhances plant response locally (around the infection sites) and systemically to alert healthy plant parts. These responses include signal transduction, synthesis of resistance-related compounds such as phytoalexins, pathogenesis-related protein (PR-protein) callose formation, lignification, and synthesis of proteinase inhibitors (Katiyar et al., 2015; Chun and Chandrasekaran, 2019). Evidence shows that chitosan also increases the endogenous 2-oxo-phytodeinoic and JA levels in many crops, including *Oryza sativa* (Rakwal et al., 2002). Furthermore, chitosan has been found responsible for activating the octadecanoic acid pathway that enhances the activity of chitinase, glucanase, and lipoxygenase and accumulates phytoalexins (El Hadrami et al., 1997; El Hadrami et al., 2010). **Figure 2** proposes a *modus operandi* for chitosan-induced changes in the soil and plant and how they influence the cellular physiology of stressed plants. Chitosan seems to have intricate crosstalk with several other signalling pathways to confer biotic tolerance. These pathways can include phytohormones (SA, JA, and ET), ROS and antioxidant metabolisms, and other cell signals.

## 4 Chitosan’s crosstalk with phytohormones, antioxidants, and other signalling molecules

### 4.1 Crosstalk with phytohormones

It is suggested that chitosan induces plant defence through an intricate correspondence with several phytohormones such as JA, abscisic acid (ABA), indole acetic acid (IAA), SA, and gibberellic acid (GA) (Jogaiah et al., 2020; Ji et al., 2022). These phytohormones are critical to inducing defence against several plant pathogens and pests.

Chitosan promotes JA concentration in plant tissues under attack by activating the octadecanoic pathway (Rakwal et al., 2002; Iriti et al., 2009). The pathway is responsible for JA biosynthesis by oxidising linolenic acid and increasing phytoalexin content. Chitosan-induced JA signalling promoted the accumulation of several other secondary metabolites such as glucosinolates and anthocyanins glucosides (Iula et al., 2022). These compounds regulate stress-induced oxidative damage given their ROS scavenging nature. Furthermore, JA can trigger signal transduction and activates defence-related genes against pathogen invasion. Peian et al. (2021) reported that chitosan activated multiple JA-biosynthesis-related genes such as *VvLOX*, VvAOC, *VvAOS*, and *VvCOI1* to induce production of methyl jasmonate, a vital defence hormone, in *Vitis vinifera* L. under fungal stress.

ABA-mediated signal transduction is critical for plants to respond to biotic and abiotic stresses (Mauch-Mani and Mauch, 2005; Lee and Luan, 2012). Chitosan is reported to stimulate ABA biosynthesis to protect the plant against *P. palmivora* in *H. brasiliensis* (Kuyyogsuy et al., 2018). In *Fragaria × ananassa* ‘Fugilia’, TOPLESS-related 3 (TPR3) and HISTONE DEACETYLASE 19 (HDA19) are overexpressed during pathogen attack. These proteins interact with the cell wall and reduce its stability. Nonetheless, both TPR3 and HDA19 were restricted by chitosan integration into such plants while restoring cell wall stability and fruit quality (Peian et al., 2021). Chitosan and ABA treatments induced the expression of *HbPR1*, *HbGLU*, *HbASI*, and *HbCAST* and the deposition of lignin and callose (Kuyyogsuy et al., 2018). ABA also regulates callose deposition intensity and speed (Flors et al., 2005). Furthermore, the same study (Kuyyogsuy et al., 2018) links chitosan with upregulated NCED activity, a crucial enzyme for ABA biosynthesis. Thus, chitosan and ABA seem to work together to defend plants against stressful scenarios. However, their precise crosstalk is still unclear. Chitosan can activate the pathogen-associated molecular pattern (PAMP) and promote H^+^ and Ca^2+^ entry in the cytosol; this activates mitogen-activated protein kinases (MAPKs) and the production of JA, ABA, and phytoalexins (El Hadrami et al., 2010; Iula et al., 2022).

In *Helianthus annuus* L., chitosan magnified IAA and phenol content during stress (Li et al., 2019; Bakhoum et al., 2020). Similarly, chitosan treatment upregulated IAA and SA content in *Arabidopsis thaliana* by altering their gene expression patterns in the roots. It resulted in restricted expression of *WOX5* in the apical root meristem and arrested root development (Lopez-Moya et al., 2017). Chitosan-induced IAA amassing could be triggered by upregulated genes of the tryptophan-dependent biosynthesis pathway (*ami1*, *aao1*, and *yuc2*) and reduced expression of IAA translocation gene (*pin1*) (Lopez-Moya et al., 2019).

Similar implications of chitosan treatment were observed in GA biosynthesis. Seed priming with chitosan improved germination rate, lipase activity, and seedling growth through increased GA levels in peanut plants (Zhou et al., 2002). Chitosan was also linked with the enhanced impact of GA on plant physiology in another study with *P. vulgaris* L. (Pereira et al., 2017). Nonetheless, the understanding of chitosan–GA crosstalk is still preliminary and needs more attention.

### 4.2 Chitosan crosstalk with ROS and antioxidant metabolism

Chitosan triggers signal transduction for phytoalexins production, secondary metabolites, and enzymatic and non-enzymatic antioxidants in defence responses to (a)biotic stresses (Pongprayoon et al., 2022). Chitosan triggered signalling pathways in strawberry fruits during oxidative stresses. It induces chloroplast-related genes. Peroxiredoxin-ROS scavenger genes have related to the cellular levels of ROS in the signalling networks of the chloroplast (Awad et al., 2015). Chitosan application in arbuscular mycorrhizal (AM) tomato (*Solanum lycopersicum* L.) promoted the growth of plants. Among the possibilities to evaluate the influence of chitosan on tomato growth and flowering may be its anti-transpirant properties to activate ROS scavenging to increase stomatal conductance and xylem vessel growth. In addition, applying chitosan to leaves increased plants’ photosynthesis rate and consequently improved plant growth and development (El Amerany et al., 2020). Plants treated with chitosan developed an increased capacity to produce enzymatic antioxidants such as CAT, POD, superoxide dismutase (SOD), and glutathione reductase (GR) to mitigate the effects of oxidative stress in salinity stress (Alkahtani et al., 2020). Chitosan’s involvement with *vide supra* specialised molecules has been reported in several plant species under various stresses. Chitosan stimulates the activity of several defence-related enzymes such as POD in *Prunus persica* L. fruits or *Phoenix dactylifera* L. roots (Abdellatef et al., 2022). Chitosan treatment can amplify PAL activity as well in many crops like *Triticum aestivum*, *V. vinifera*, and *O. sativa*, resulting in increased levels of phenolic and flavonoid compounds through phenylpropanoid pathway (Li et al., 2013). In *V. vinifera*, chitosan elevated PAL activity and enhanced the antioxidant defence mechanisms against *Botrytis cinerea* by upregulated CAT, POD, and SOD activities (Peian et al., 2021).

The foliar application of chitosan encouraged O_2_^−^ scavenging and restricted H_2_O_2_ generation and lipid peroxidation to manage stress in sweet peppers (Alkahtani et al., 2020). In white clover, chitosan in dehydration-responsive element-binding protein (DREB) responsive pathway upregulated *DREB2*, *DREB4*, and *DREB5* genes (Ling et al., 2022). It activated *Y2K* and *Y2SK* genes, which encode dehydrins (DHNs) that produce water-stress tolerance. Notably, these genes are critical to stress tolerance and antioxidant defence.

### 4.3 Chitosan crosstalk with signalling molecules

Chitosan activates signalling pathways in cells by binding to specific cellular receptors, activating important secondary messengers such as Ca^2+^, nitric oxide (NO), ROS, and transcription factors (TFs) (*vide supra* section 4.2 for ROS). These molecules play a critical role in triggering several biochemical responses. It is worth noting that chitosan, along with Ca^2+^ treatment, increases Ca^2+^ influx into the cytosol. This elevated cytosolic Ca^2+^ level is associated with enhanced callose formation. Notably, chitosan without the Ca^2+^ application did not form callose, suggesting that chitosan-induced callose synthesis depends on the presence of Ca^2+^ (Koühle et al., 1985).

Similarly, NO plays a critical role in diverse vital physiological phenomena and provides defence against stress scenarios (Corpas et al., 2001; Kolbert et al., 2019). Chitosan promotes the generation of NO and phosphatidic acid. Nonetheless, it inhibits the phospholipase-mediated signalling pathway in the presence of an NO scavenger. Thus, it seems plausible that chitosan elicits defence responses in a NO-dependent pathway (Tocci et al., 2011). Moreover, chitosan can regulate photosynthesis and stomatal movement (Mukarram et al., 2023) in NO-dependent signalling, considering NO has critical roles in stomatal movement in stressed plants (Garcıía-Mata and Lamattina, 2001; Neill et al., 2008).

Many chitosan-modulated genes, including defence-related genes and TFs related to signalling pathways, are involved in biotic stress responses. Povero et al. (2011) demonstrated that several WRKY genes of *A. thaliana* respond to chitosan treatment. WRKY gene family is attributed to defending against pathogens or pathogen-mimicking stimuli. Among these chitosan-elicited WRKY TFs, At5g13080 (WRKY75), At3g01970 (WRKY45), At2g46400 (WRKY46), and At4g31800 (WRKY18) are specifically involved in pathogen responses. Chitosan also overexpressed other MYB TFs such as MYB31 (At1g74650) and MYB15 (At3g23250). The modulation of these TFs by chitosan highlights its impact on regulating biotic stress responses and emphasises its potential as a valuable tool in enhancing plant defence mechanisms.

## 5 Technological advances: chitosan oligomers (COS), chitosan microparticles (CS-MPs), and chitosan nanoparticles (CS-NPs)

At high pH (≥6.5), chitosan experiences reduced solubility, high viscosity, and affinity to coagulate proteins. This limits chitosan’s bioactivities. The radiolytic degradation or (acidic, alkaline, or enzymatic) digestion of the β-1,4-glycosidic bonds between monomeric sugar residues in chitosan polymers can form chitosan oligosaccharides (COSs). Such oligosaccharides have higher solubility and surface area and lower viscosity than their polymeric counterparts (Muley et al., 2019). Thus, COSs are equipped with upgraded bioactivities, e.g., antimicrobial (against fungi, bacteria, and viruses), antitumor, antioxidant, anti-inflammatory, hypocholesterolemic, and immunopotentiation bioactivities (Liaqat and Eltem, 2018). Similarly, COS application in agriculture produced superior outcomes in plant growth, development, productivity, and defence against (a)biotic stresses (Wang et al., 2015; Lan et al., 2016; Li et al., 2020; Mukarram et al., 2022). Kim and Rajapakse (2005) reported that COSs discourage pathogenic invasion on the plant by upregulating various genes expression responsible for endogenous plant immunity. It was suggested that poly-d-glucosamine units of chitosan bind with the contagious receptors mimicking a pathogenic invasion (Maurya et al., 2019). It initiates the feedback mechanisms in the plant including upregulated phytoalexins biosynthesis. Phytoalexins are the defence chaperones for endogenous immunity and confer resistance against biotic stress. Others reported a boost in phytoalexin content with COS application in different plants (Pichyangkura and Chadchawan, 2015). Benchamas et al. (2021) discussed antibiotic effects of COSs against several gram (+) bacteria (e.g., *M. luteus*, *Staphylococcus faecalis*, *S. aureus*, *B. subtilis*, *B. cereus*, and *Lactiplantibacillus plantarum*) and gram (−) bacteria (such as *E. coli*, *Aggregatibacter actinomycetemcomitans*, *Vibrio vulnificus*, and *P. aeruginosa*). Similar cytotoxic effects of COSs were found in many important fungi, e.g., *Candida albicans*, *Candida krusei*, *B. cinerea*, *Saccharomyces cerevisiae*, *Rhodotorula glutinis*, *Rhodotorula mucilaginosa*, and *Schizosaccharomyces pombe* (Ganan et al., 2019).

Chitosan microparticles (CS-MPs) or nanoparticles (CS-NPs) are even smaller chitosan derivatives than COSs. CS-MPs are produced from the aqueous solution of chitosan mixed with dilute acid. The resulting suspension loses its aqueous phase at low pressure and forms microparticles. CS-MPs formed from low chitosan concentration can stabilise Pickering emulsion (oil-in-water) (Mwangi et al., 2016). This opens new perspectives for optimising stimulus-responsive emulsion and their stable storage. Several studies with CS-MPs or CS-NPs concluded their enhanced beneficial role in agriculture, food storage, and biomedical sectors over chitosan polymers (Mwangi et al., 2016; Iglesias et al., 2019; Malerba and Cerana, 2020). Further, CS-MPs and CS-NPs have special relevance in vaccine delivery, inflammatory diseases, and cancer treatment (see Prabaharan and Mano, 2004; Naskar et al., 2019). These bioactivities could also be true against microbial and pest communities. CS-NPs exhibit cytotoxic effects against many gram (+) and gram (−) bacteria, e.g., *E. coli*, *Staphylococcus choleraesuis*, *Staphylococcus typhimurium*, and *S. aureus* (Qi et al., 2004). Ahmed and Aljaeid (2016) suggested the crucial roles of CS-MPs and CS-NPs with the influenza vaccine, cholera toxin, hepatitis B surface protein, and antigen protein against several other fungi and viruses. Integrating CS-NPs with certain metals, phenolics, and essential oils produces enhanced antioxidants and scavenging activities against microbes (Hasheminejad et al., 2019; Fahimirad et al., 2021; Rashki et al., 2021). This makes chitosan particles an ideal candidate for encapsulating agents in the food packaging and preservation industry as well as a delivery vehicle for bioactive compounds such as nutrients, essential oils, vitamins, and antioxidants (Hasheminejad et al., 2019; Maleki et al., 2022).

## 6 Conclusion and perspectives

The past few decades have witnessed exponential growth in chitosan studies in several aspects of crop defence to biomedical applications. It is established now that chitosan boosts plant development and yield during optimal and stressful environments. Nonetheless, there is a vast gap in the molecular understanding of chitosan and its derivatives. In particular, a comprehensive cognition of chitosan’s crosstalk with phytohormones, antioxidants, and signalling molecules is lacking. It is pertinent for future studies to explore the signalling potential of chitosan itself. Although our knowledge of chitosan’s antimicrobial potential has widened over the past two decades, multiple inconsistencies and a well-defined mechanism must be solved. Therefore, another aspect worth exploring is the *modus operandi* of chitosan against microbial entities such as fungi, bacteria, and viruses. Recent advances in structural modification in chitosan conferred superior results over chitosan. It could be interesting to know whether these modified chitosan oligomers or nanoparticles adopt different pathways or signalling molecules for enhanced bioactivities.

## Author contributions

All authors listed have made a substantial, direct, and intellectual contribution to the work and approved it for publication.
